# Mindfulness and Suicide Risk in Undergraduates: Exploring the Mediating Effect of Alexithymia

**DOI:** 10.3389/fpsyg.2019.02106

**Published:** 2019-09-13

**Authors:** Yuan Fang, Baoer Zeng, Peiyi Chen, Yiling Mai, Shan Teng, Minting Zhang, Jingbo Zhao, Xueling Yang, Jiubo Zhao

**Affiliations:** ^1^Department of Psychology, School of Public Health, Southern Medical University, Guangzhou, China; ^2^Department of Psychiatry, Zhujiang Hospital, Southern Medical University, Guangzhou, China

**Keywords:** suicide, suicide risk, mindfulness, alexithymia, gender differences, undergraduates

## Abstract

The present study was designed to examine the relationship between dispositional mindfulness and suicide risk in undergraduates, and it further explored the potential mediating role of alexithymia in this relationship. A total of 2,633 undergraduates completed the Mindful Attention Awareness Scale (MAAS), the Suicidal Behaviors Questionnaire – Revised (SBQ-R), and the 20-item Toronto Alexithymia Scale (TAS-20). The results indicate that mindfulness and suicide risk were negatively correlated, and alexithymia partially mediated the relationship between mindfulness and suicide risk only in the female undergraduates. Moreover, only the difficulty in identifying feelings (DIF) factor of alexithymia mediated the relationship between mindfulness and suicide risk in the female undergraduates. These findings contribute to the potential mechanism that explains the relationship between mindfulness and suicide risk. Furthermore, it is possible to implement mindfulness in the suicide intervention of alexithymic individuals.

## Introduction

Suicidal behavior is a significant global public health problem that accounts for over 800,000 deaths each year (World Health Organization [WHO], 2014). In China, suicide is the second leading cause of unnatural deaths among undergraduates (Yang and Li, 2015). A multinational study indicated that the past 1-year prevalence of suicidal ideation, plans, and attempts in undergraduates were 17.2, 8.8, and 1.0%, respectively (Mortier et al., 2018). Under this background, it is important to understand the modifiable risk factors associated with suicide in the undergraduate population, as this may provide guidance for college students’ suicide intervention and reduce the risk of suicide among college students. Emotional problems have always been an important factor in suicide (Pompili, 2010). Some scholars believe that suicide is an extreme coping style adopted by individuals in the face of significant pressure who are unable to effectively regulate negative emotions (Neece et al., 2013). The perception and identification of emotions are the bases for emotional regulation (Taylor et al., 1997). Therefore, individuals who have difficulty in identifying emotions (e.g., alexithymic individuals) may have a higher suicide risk.

### Alexithymia and Suicide Risk

Alexithymia is conceptualized as a difficulty in identifying, processing, and describing emotions (Sifneos, 1973). The salient features include difficulty identifying feelings, difficulty describing feelings, and an externally oriented cognitive style (Bagby et al., 1994). Individuals who are unaware of their feelings often suffer from emotional dysregulation, specifically, the deficits in experience, acceptance and influence of emotions, which is one of the most important risk factors for suicide (Gratz and Roemer, 2004; De Berardis et al., 2008). When facing stressful events, alexithymic individuals may have more difficulty reducing negative-emotion arousal and have a higher suicide risk. Theories about the potential emotional process of suicide (Baumeister, 1990; Williams, 2001) suggest that alexithymia plays an important role in suicidality. Shneidman (1984) posited that suicide is a way for hopeless individuals to escape from unbearable emotion and anguish. Similarly, the Cry of Pain model (Williams, 2001) proposes that suicide is a “cry of pain” response when individuals feel defeated and cannot escape. According to the model, individuals engage in suicidal behavior when they believe that negative emotions are unbearable, especially when they are unable to effectively regulate emotion because of the difficulty in identifying feelings. Taken together, contemporary suicide-focused theories implicate the important role of alexithymia in impacting suicidal symptoms.

In fact, empirical studies have shown that alexithymia may be positively correlated with higher levels of suicide risk (De Berardis et al., 2017). For example, researchers have found that alexithymia was associated with a higher suicide risk in Obsessive-Compulsive Disorder (OCD) patients (De Berardis et al., 2014). Specifically, among college students, alexithymia was also found to have a positive association with suicidal ideation (Feng et al., 2016). Moreover, there is substantial evidence that alexithymia predicts the onset and maintenance of suicidal ideation. In a 12-month follow-up study, Hintikka et al. (2004) found that a decrease in alexithymia during the study period was associated with recovery from suicidal ideation, while an increase in alexithymia was associated with the occurrence of suicidal ideation.

### Mindfulness, Alexithymia and Suicide Risk

Considering the relation between alexithymia and suicide risk, it is necessary to study the factors that have a positive effect on alexithymia. Mindfulness has proven to be efficacious in the treatment of emotional problems (Britton et al., 2012; Santarnecchi et al., 2014**)**. Mindfulness originated in the traditions of Buddhism and is defined as an awareness of the present experience with an attitude of acceptance and non-judgment (Kabat-Zinn, 1994). The essence of mindfulness, present moment awareness, not only can be cultivated through mindfulness-based training but also may exist as an individual disposition (Brown and Ryan, 2003). Interestingly, the characteristics of mindfulness can be contrasted with alexithymia. People with alexithymia show an externally oriented cognitive style and lack of attention to their inner world (Taylor, 2000). By contrast, mindfulness encourages observation and acceptance of thoughts, feelings and sensations, which in turn leads to a greater understanding of the feelings as they occur (Coffey et al., 2010). A substantial body of literature has documented the positive effects of mindfulness on alexithymia. In a survey of college students, Macdonald and Price (2017) found that alexithymia was inversely related to mindfulness. Furthermore, the results of an intervention study found decreased alexithymia in individuals who undergo mindfulness training, and this change was correlated with neuroanatomical changes (Santarnecchi et al., 2014).

In addition, the positive effects of mindfulness on suicide have also been well documented in many studies. For example, in a survey of college students, Lamis and Dvorak (2014) found that dispositional mindfulness was negatively associated with suicidal ideation. Furthermore, interventions based on mindfulness, such as mindfulness-based cognitive treatment (MBCT), may help reduce an individual’s suicidal risk (Williams et al., 2006). In support of this, Chesin et al. (2015) implemented a MBCT clinical intervention study, and the results indicated that MBCT was effective in reducing suicidal ideation among outpatients who presented an elevated suicide risk.

Accordingly, considering the robust relation between alexithymia and suicide risk and the significant influence of mindfulness on alexithymia, it is reasonable to assume that alexithymia may mediate the relation between mindfulness and suicide risk. However, no study has examined the role of alexithymia in the relationship between mindfulness and suicide risk.

### The Current Study

The primary purpose of the current study was to examine the mediating role of alexithymia in the relationship between mindfulness and suicide risk. As there are known sex differences in suicide (e.g., Dai et al., 2011), the possible moderating role of the participant’s sex also needs to be controlled for in the analysis. Based on studies that show the relationships of alexithymia with suicide risk (e.g., Hintikka et al., 2004; De Berardis et al., 2014), mindfulness with alexithymia (e.g., Arias et al., 2010; Macdonald and Price, 2017), and mindfulness with suicide risk (e.g., Lamis and Dvorak, 2014; Chesin et al., 2015), we proposed the following hypotheses: (1) Mindfulness is negatively associated with suicide risk. (2) Mindfulness is negatively associated with alexithymia. (3) Alexithymia is positively associated with suicide risk. (4) Alexithymia may mediate the association between mindfulness and suicide risk. (5) The mediating effect of alexithymia may be different across genders.

## Materials and Methods

### Participants and Procedure

In total, 2,953 freshmen from a medical university in China volunteered to participate in this study, and 2,633 of these participants (89.2%) completed the entire survey. Prior to conducting the study procedures, informed consent was obtained from all participants. They were requested to complete a package of online questionnaires in school computer labs at a prescribed time. The Ethics Committee approved the study in advance of the data collection.

In our sample, 60.9% of the participants were female (*M*_age_ = 18.25, *SD* = 0.87), and 39.1% were male (*M*_age_ = 18.34, *SD* = 0.98), with an age range from 15 to 23 years.

### Measures

#### Dispositional Mindfulness

The Mindfulness Attention and Awareness Scale (MAAS; Brown and Ryan, 2003) was used to assess dispositional mindfulness. The MAAS is a self-reported measure that assesses dispositional mindfulness. The scale consists of 15 items rated on a 6-point scale from 1 (almost always) to 6 (almost never). Higher scores indicate greater levels of dispositional mindfulness. This instrument has been validated in college students (α = 0.82) (Brown and Ryan, 2003). Furthermore, the Chinese version of the MAAS has demonstrated high reliability among undergraduates (α = 0.89) (Chen et al., 2012). In the present sample, the Cronbach’s α of the MAAS was 0.90.

#### Suicide Risk

Suicide risk was assessed by using the Suicidal Behaviors Questionnaire – Revised (SBQ-R; Osman et al., 2001). The SBQ-R consists of four items that assess lifetime suicidal ideation and attempts, the frequency of suicidal ideation over the past year, the threat of suicidal behavior, and the self-reported likelihood of future suicidal behavior. The total score ranges from 3 to 18, and a cutoff score of 7 for the general population can be used to determine clinically significant levels of suicide risk (Osman et al., 2001). Higher scores indicate a higher suicide risk. The reliability and validity of the SBQ-R have been validated (Osman et al., 2001). The Chinese version of the scale has also demonstrated high reliability among college students (α = 0.68) (Zhao et al., 2012). In the present sample, the Cronbach’s α of the SBQ-R was 0.69.

### Alexithymia

Alexithymia was measured by using the self-reported 20-item Toronto Alexithymia Scale (TAS-20; Bagby et al., 1994). This scale assesses alexithymia based on the following three dimensions: difficulty in identifying feelings (DIF), difficulty in describing feelings (DDF) and externally oriented thinking (EOT). Each item is answered on a five-point Likert scale that ranges from strong disagreement to strong agreement. Higher TAS-20 scores reflect higher alexithymia levels. The Chinese version of the TAS-20 has high internal consistency among undergraduates (Cronbach’s α = 0.83) (Yi et al., 2003). In the present sample, the subscales DIF and DDF showed Cronbach’s alphas of 0.85 and 0.68, respectively. The subscale EOT showed an alpha of 0.54 and was excluded from the analysis. The alpha for the total scale was 0.85.

### Data Analyses

First, descriptive statistics were used to describe the data. The categorical variables (e.g., gender) are reported as percentages, and the continuous variables (e.g., age) are reported as the mean ± standard deviation. Gender differences were assessed by using *t* tests and a chi-square test. A Pearson correlation was used to examine the relationships among the MAAS, SBQ-R, and TAS-20. Then, AMOS 24.0 software was used for a mediation analysis that employed structural equation modeling (SEM). The following fit indexes were used to examine the model fit (Hu and Bentler, 1999): (a) the root mean square error of approximation (RMSEA), with values of 0.08 or less reflecting a reasonable fit; (b) the comparative fit index (CFI), with values of 0.90 and higher indicating a good fit; (c) the goodness of fit indicator (GFI), with values of 0.90 and higher reflecting a good fit; (d) and the normed fit index (NFI), with values of 0.90 and higher indicating a good fit. Bootstrapping procedures were adopted to test the significance of the mediated model. In this study, we generated 5,000 bootstrapping samples from the original data set (*N* = 2633) by random sampling, and a *p*-value of 0.05 was set as the critical level for statistical significance.

## Results

The means, standard deviations (SDs), and correlations among the study variables are reported in Table 1 together with the proportions of the participants whose scores exceeded the clinically significant cut-off values. Approximately 13.5% of the participants scored at or above the SBQ-R cut-off score of 7. Females had significantly higher total SBQ-R scores than males and were also more likely to be classified with suicide risk (15.5 and 10.5%, respectively). In addition, significant gender differences were found in the DIF factor of the TAS-20 (15.12 ± 4.8 for males, 15.54 ± 4.3 for females, *P* < 0.001). There was no difference in the MAAS, TAS-20 total score, and DDF of the TAS-20 between males and females.

**TABLE 1:** Correlations, means, standard deviations of measured variables.

**Measures**	**(2)**	**(3)**	**(4)**	**(5)**	**Total sample**	**Male**	**Female**
					(*N* = 2633)	(*N* = 1030)	(*N* = 1603)
					M ± SD	M ± SD	M ± SD
(1)Mindfulness	−0.64^∗^	−0.61^∗^	−0.57^∗^	−0.24^∗^	67.48 (11.30)	67.53 (12.19)	67.46 (10.70)
(2)Alexithymia		0.89^∗^	0.86^∗^	0.20^∗^	46.02 (9.28)	45.77 (9.95)	46.18 (8.82)
(3)DIF			0.74^∗^	0.23^∗^	15.38 (4.50)	15.12 (4.82)	15.54 (4.23) ^∗^
(4)DDF				0.19^∗^	12.29 (3.21)	12.36 (3.47)	12.24 (3.03)
(5)Suicide Risk					4.54 (1.90)	4.34 (1.75)	4.68 (1.98) ^∗^
					N (percent)	N (percent)	N (percent)
SBQ-R ≥ 7					356 (13.5%)	108 (10.5%)	248 (15.5%)^∗^

*^∗^*p* < 0.01; DIF: Difficulty in identifying feelings; DDF: Difficulty in describing feelings.*

As shown in Table 1, dispositional mindfulness was negatively correlated with alexithymia and suicide risk. A significant positive association was found between alexithymia and suicide risk. The DIF and DDF dimensions of alexithymia were negatively correlated with dispositional mindfulness and positively correlated with suicide risk. These significant intercorrelations also existed in males and females (see Table 2). However, these results must be carefully interpreted, as the sample in this study is considerable.

**TABLE 2:** Correlations, means, standard deviations of measured variables.

**Measures**	**(1)**	**(2)**	**(3)**	**(4)**	**(5)**
(1)Mindfulness		−0.68^∗^	−0.66^∗^	−0.62^∗^	−0.29^∗^
(2)Alexithymia	−0.60^∗^		0.91^∗^	0.87^∗^	0.22^∗^
(3)DIF	−0.57^∗^	0.89^∗^		0.76^∗^	0.24^∗^
(4)DDF	−0.52^∗^	0.85^∗^	0.73^∗^		0.22^∗^
(5)Suicide Risk	−0.22^∗^	0.19^∗^	0.22^∗^	0.18^∗^	

*^∗^*p* < 0.01; DIF: Difficulty in identifying feelings; DDF: Difficulty in describing feelings; Zero-order correlations are presented above the diagonal for male (*n* = 1030); for female (*n* = 1603), below the diagonal.*

**FIGURE 1:**
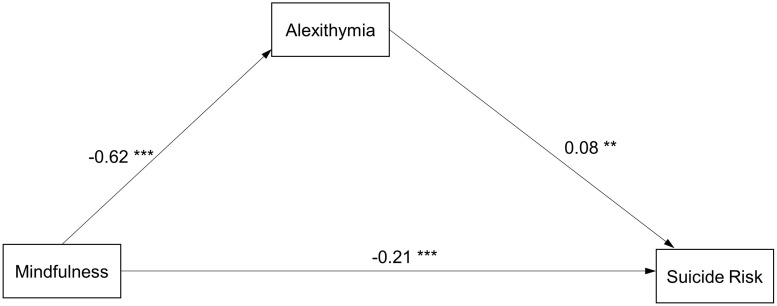
Path diagram with structural equation modeling results and standardized path coefficients in all samples (Model 1). ^∗^*p* < 0.05; ^∗∗^*p* < 0.01; ^∗∗∗^*p* < 0.001 (Model fit: X2/df = 8.198, *p* < 0.001, RMSEA = 0.052, CFI = 0.949, GFI = 0.982, NFI = 0.942).

**FIGURE 2:**
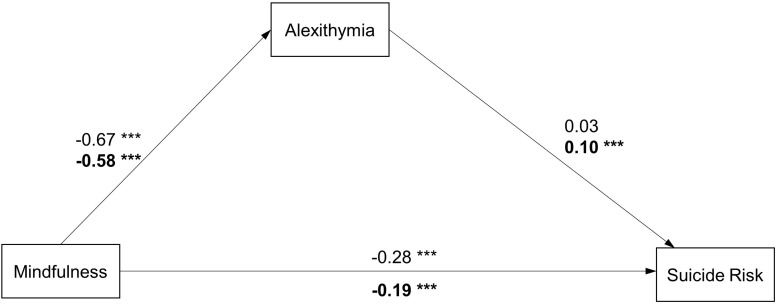
Path diagram with structural equation modeling results and standardized path coefficients in males/females (Model 1). ^∗^*p* < 0.05; ^∗∗^*p* < 0.01; ^∗∗∗^*p* < 0.001 Model fit: Males: x2/df = 2.540, *p* < 0.001, RMSEA = 0.039, CFI = 0.980, GFI = 0.979, NFI = 0.924; Females: x2/df = 5.577, *p* < 0.001, RMSEA = 0.053, CFI = 0.944, GFI = 0.980, NFI = 0.933 Note: coefficients for females are presented in bold.

**FIGURE 3:**
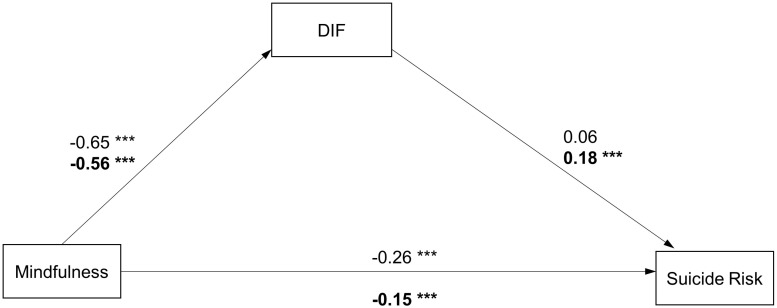
Path diagram with structural equation modeling results and standardized path coefficients in males/females (Model 2). ^∗^*p* < 0.05; ^∗∗^*p* < 0.01; ^∗∗∗^*p* < 0.001 Model fit: Males: x2/df = 3.498, *p* < 0.001, RMSEA = 0.049, CFI = 0.960, GFI = 0.981, NFI = 0.945; Females: x2/df = 5.379, *p* < 0.001, RMSEA = 0.052, CFI = 0.941, GFI = 0.980, NFI = 0.930 Note: coefficients for females are presented in bold.

**FIGURE 4:**
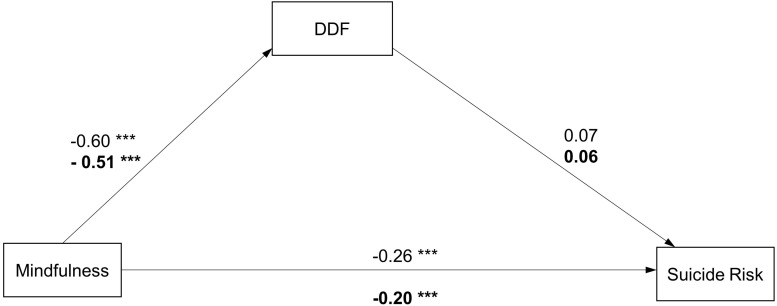
Path diagram with structural equation modeling results and standardized path coefficients in males/females (Model 3). ^∗^*p* < 0.05; ^∗∗^*p* < 0.01; ^∗∗∗^*p* < 0.001. Model fit: Males: x2/df = 3.515, *p* < 0.001, RMSEA = 0.049, CFI = 0.954, GFI = 0.982, NFN0.937; Females:x2/df = 6.982, *p* < 0.001, RMSEA = 0.061, CFI = 0.916, GFN0.976, NFN0.905 Note: coefficients for females are presented in bold.

Figure 1 depicts a mediation pathway diagram that reflects the SEM results in all participants (Model1). The model had a good data fit (χ^2^/df = 8.198, *p* < 0.001, RMSEA = 0.052, CFI = 0.949, GFI = 0.982, NFI = 0.942). The structural pathways were consistent with the hypothesized directions. All pathways in the model were significant. More importantly, bootstrap analyses showed that the relationship between mindfulness and suicide risk was partially mediated by alexithymia (with the indirect effect = −0.05, CI[−0.09, −0.01]). Figures 1–4 show the path coefficients of the model, and the numbers in the figures are the beta-weights. The indirect effects of mindfulness on suicide risk are calculated by taking the product of the two beta-weights in the two arms of the diagram that represent the mediating effect of alexithymia/factors of alexithymia.

Furthermore, separate analyses were conducted to investigate whether the model fitted well across genders (Model 1). As shown in Figure 2, The model had a good fit in both groups (male/female: χ^2^/df = 2.540/5.577, *p* < 0.001 for both, RMSEA = 0.039/0.053, CFI = 0.980/0.944, GFI = 0.979/0.980, NFI = 0.924/0.933). All pathways of the model in the female participants were significant. However, the pathway from the TAS-20 to the SBQ-R in the male participants was not significant (β = 0.03, CI[−0.07, 0.14], *p* = 0.509).

In addition, separate analyses were conducted to investigate which factor of the TAS-20 plays a mediating role between mindfulness and suicide risk in males and females. As shown in Figures 3, 4, all the models had a good fit in both groups (Model 2 and Model 3). In the male group, the pathways from the DIF to the SBQ-R and from the DDF to the SBQ-R were not significant (β = 0.06, CI[−0.05, 0.19], *p* = 0.247; β = 0.07, CI[−0.03, 0.19], *p* = 0.125). In the female group, only the pathway from the DDF to the SBQ-R was not significant (β = 0.06, CI[−0.01, 0.14], *p* = 0.089). The results indicate that the DIF factor significantly mediated the association between mindfulness and suicide risk in the female group (the indirect effect = −0.10, CI[−0.15, −0.06]). The direct and indirect effect values for all models are shown in Table 3.

**TABLE 3:** The paths and effect analysis.

	**Effect**	**Paths**	**Effect size**		
			**Total sample**	**Male**	**Female**
Model1	Direct effect	Mindfulness→Suicide risk	−0.21^a^	−0.28^a^	−0.19^a^
	Indirect effect	Mindfulness→Alexithymia→Suicide risk	−0.05^a^	−0.02	−0.06^a^
Model2	Direct effect	Mindfulness→Suicide risk	–	−0.26^a^	−0.15^a^
	Indirect effect	Mindfulness→DIF→Suicide risk	–	−0.04	−0.10^a^
Model3	Direct effect	Mindfulness→Suicide risk	–	−0.26^*a*^	−0.20^a^
	Indirect effect	Mindfulness→DDF→Suicide risk	–	−0.04	−0.03

*^*a*^95% CI does not overlap with zero (*p* < 0.05); DIF: Difficulty in identifying feelings; DDF: Difficulty in describing feelings.*

## Discussion

The current study examined suicide risk among undergraduates and tested a mediation model in which alexithymia mediated the relationship between mindfulness and suicide risk. Approximately 13.5% of the participants reported a non-clinical suicide risk. This rate is generally less than the rates reported in a large sample survey of undergraduates (Farabaugh et al., 2015; Becker et al., 2018a). This discrepancy may be due to the fact that our sample consists entirely of freshmen who just entered the university. Compared with college students at other levels, freshmen are still curious about new college life and face less stress (such as interpersonal stress, academic stress or employment pressure) and are less likely to have psychological problems (Hu, 2018).

In our study, the results show that females were more likely to be classified with suicide risk than males, which is consistent with previously research (Becker et al., 2018b; Mirandamendizabal et al., 2019). A possible explanation for this difference might be that female students are more sensitive than male students, and they are more prone to emotional problems, thereby increasing their risk of suicide (Kaess et al., 2011). Another explanation is that females are under greater psychological stress because of their tendency to focus on feelings of personal weakness and inferiority (i.e., blaming themselves) when they get frustrated (Martínez et al., 2014). There were non-significant gender differences in alexithymia (total TAS-20 and the DDF factor) in the same vein as previous research (Kiselica and O’ Brien, 2001; De Berardis et al., 2013). Gender differences were found in the DIF factor of alexithymia, with female students showing higher scores, which contrasts with research that indicates that males have higher levels of alexithymia than females (Chen and Chung, 2016). Nevertheless, our finding is consistentwith other studies that show the converse (Levant et al., 2009; Viinikangas et al., 2009). The magnitude of the difference was small but indicates that females may have more difficulties in identifying their feelings. In addition, with the present sample, no significant gender difference was found in mindfulness, which suggests that being mindful is an important protective factor of suicide for both males and females, which is in line with the results of previous research (Brown and Ryan, 2003; Howell et al., 2008).

The correlational analysis shows that mindfulness was negatively associated with alexithymia and suicide risk. These findings are in line with former research that reported relationships between mindfulness and alexithymia (e.g., Arias et al., 2010; Macdonald and Price, 2017) and between mindfulness and suicide risk (e.g., Lamis and Dvorak, 2014; Chesin and Jeglic, 2016). Moreover, we found a positive association between alexithymia and suicide risk, which parallel the findings from previous research (e.g., Hintikka et al., 2004; De Berardis et al., 2014). The DIF and DDF dimensions of alexithymia show significant positive correlations with suicide risk and negative correlations with mindfulness. All of these findings hold across both the full sample and by sex separately.

In accordance with our expectations, alexithymia mediated the association between mindfulness and suicide risk, which is in the same vein with suicide-related theory and previous studies. One way to appropriately regulate negative emotions is by being able to identify and describe what one is feeling. Alexithymic individuals have difficulties in identifying or describing their feelings; thus, they may turn to suicide as a way to escape their negative emotions. This may be especially the case when the stressors are significant and persistent (for example, interpersonal conflict). Unfortunately, alexithymic individuals are more likely to elicit interpersonal conflict because their empathy is impaired and they may not be able to respond effectively in the absence of the ability to identify and describe feelings (Lumley et al., 1996; Bird and Viding, 2014). Mindfulness, the awareness of one’s internal experience (thoughts, bodily sensations and feelings) in the present moment, could promote individuals’ ability to identify and describe their feelings, which is the foundation of emotion regulation (Arch and Craske, 2006). When facing stressful events, mindful individuals, who have higher emotional regulation abilities, have less sensitivity to psychological difficulties and can recover more rapidly from negative emotions; accordingly, they have less subsequent psychological impairment (Hulsheger et al., 2013; Siman et al., 2015) and, have a lower suicide risk (Pisani et al., 2013). The idea that suicide may arise as an emotion regulation strategy due to individuals having difficulties identifying and describing feelings is similar to the escape theory of suicide (e.g., Baumeister, 1990) and with suicide intervention methods, such as Dialectical behavior therapy (DBT) and MBCT for suicidal individuals (Rathus and Miller, 2002; Williams et al., 2006). Both of these suicide intervention methods decrease suicide risk mainly by improving individuals’ emotional awareness and mitigating emotional dysregulation. Therefore, theoretical and empirical research, appear to be consistent with the current finding of alexithymia being a key factor in the relationship between mindfulness and suicide risk.

In addition, multiple additional findings remain important to discuss. Separate analyses found that the mediating effect of alexithymia is significant only in female students, which may indicate that different mechanisms are involved in the relationship between mindfulness and suicide risk between males and females. In males, the path from alexithymia to suicide risk was non-significant. These differences may be accounted for by gender socialization, where distressed females are more prone to adopt emotion-focused coping strategies, and distressed males are more prone to adopt problem-oriented coping strategies (Chan, 1994; Stanton et al., 1994). Higher levels of alexithymia represent deficits in the way that women usually cope with stress and solve problems (by identifying/describing feelings) and are therefore related to a higher suicide risk (Gabriel et al., 2016). Accordingly, decreasing alexithymia by promoting mindfulness has effectivity in the reduction of the suicide risk in females. In contrast, emotional recognition and description ability may generally play a less important role in problem solving in males (Gabriel et al., 2016); thus, the improvement of these abilities may not influence a decrease in suicide risk.

Somewhat surprisingly, further analyses showed that only the DIF dimension of alexithymia significantly mediated the relationship between mindfulness and suicide risk in females. The path from DDF to suicide risk is non-significant in females. This converges with the view that the specific dimension of alexithymia has a unique influence on specific mental symptoms (O’ Brien et al., 2008). Increased mindfulness is associated with a decreased difficulty in identifying feelings, which is, in turn, relates to a reduced suicide risk. This may be related to the role of the insula. The insula is a brain region related to emotional recognition and emotional experience. It is responsible for receiving internal body state information and integrating this information into the consciousness of feelings (Tsuchiya and Adolphs, 2007; Craig, 2009). Magnetic resonance imaging (MRI) studies suggest that there are abnormalities in the function or structure of the insula in alexithymic individuals as well as suicidal individuals. Researchers found that lower activity in the insula of individuals with high levels of alexithymia than that of individuals with low levels of alexithymia (Reker et al., 2010; Deng et al., 2013). In addition, a reduction in grey matter (GM) volume in the insula was found in subjects with alexithymia, and GM volume in the insula was negatively associated with TAS-20 scores (Ihme et al., 2013; Grabe et al., 2014). Similar findings are also present in suicidal individuals. Compared to non-attempters, suicide attempters had significantly less gray matter in insula (Giakoumatos et al., 2013). Interestingly, Haase et al. (2015) found increased insula activation during the post-load condition in individuals engaging in a mindfulness practice, and this change was correlated with an increased ability to identify feelings on the TAS-20. More importantly, a correlation between the increase in the insula thickness and the decrease in alexithymia levels after a mindfulness stress reduction program (MBSR) was also observed (Santarnecchi et al., 2014). These studies above suggest that mindfulness may improve one’s ability to identify feelings by changing the function and structure of the insula, thereby improving mental symptoms. Some scholars have indicated that improving individuals’ ability to identify feelings is an important protective factor for maintaining mental health in stressful situations (Ng and Hurry, 2011). Moreover, although the DDF did not act as a mediator, it was significantly correlated with suicide risk. This remains a critical question and important topic for future research.

Several limitations of the present study should be noted. First, the sample in our study included only undergraduates, which limits the external validity and generalizability of the results to other general populations. Thus, caution is warranted when generalizing the current findings to the general population. Second, causal relationships cannot be established given the cross-sectional design of this research; therefore, future studies should implement a mindfulness-based intervention program to clarify the directional effects. Third, the measure of mindfulness in the present study is unidimensional; it is necessary to study the multidimensional operationalization of mindfulness. Future research could further clarify which dimensions of mindfulness correlated with suicide risk and alexithymia. In addition, alexithymia is not the only mediator in the association between mindfulness and suicide risk, and mindfulness itself may have a direct association with suicide risk in females. Furthermore, alexithymia does not play a mediating role in the relationship between mindfulness and suicide in males. Future studies could include other potential mediators to reveal the mechanisms of mindfulness in suicide intervention in males and females.

Accordingly, this study indicates that dispositional mindfulness has positive influences on individuals’ suicide risk and that these effects are achieved via different pathways across genders. In female undergraduates, these effects are partially achieved through DIF factor of alexithymia. However, in male undergraduates, the mediating role of alexithymia has not been established, and mindfulness has a direct effect on suicide risk. These findings reveal the possible mechanism by which mindfulness may influence an individual’s suicide risk and provide empirical evidence for the implementation of mindfulness in suicide prevention. The cultivation of mindfulness can be used as a preventive therapy to help Chinese undergraduates decrease their levels of alexithymia and suicide risk. For example, we can combine mindfulness with music therapy, which may have the potential to achieve significant results in treating alexithymia (Allen and Heaton, 2010). After using the music to trigger an individual’s emotions, guiding the individual to practice mindfulness may be more effective in improving the individual’s emotional awareness, thereby reducing the risk of suicide. Furthermore, these results suggest that clinicians and mindfulness training indicators can directly and explicitly concentrate on alexithymia in the mindfulness-based treatment of Chinese female students to maximize the benefits of these interventions. On this basis, it is also possible to develop a mindfulness-based suicide intervention that targets alexithymia.

## Data Availability

The datasets generated for this study are available on request to the corresponding author.

## Ethics Statement

This study was carried out in accordance with the recommendations of the ethics committee for psychological research at our university. All subjects gave written informed consent in accordance with the Declaration of Helsinki. The protocol was approved by the Ethics Committee of Southern Medical University.

## Author Contributions

JiuZ designed the study. All coauthors are participants in the data collection and analysis, writing, and revising the manuscript.

## Conflict of Interest Statement

The authors declare that the research was conducted in the absence of any commercial or financial relationships that could be construed as a potential conflict of interest.
